# Identification of positively selected genes in human pathogenic treponemes: Syphilis-, yaws-, and bejel-causing strains differ in sets of genes showing adaptive evolution

**DOI:** 10.1371/journal.pntd.0007463

**Published:** 2019-06-19

**Authors:** Denisa Maděránková, Lenka Mikalová, Michal Strouhal, Šimon Vadják, Ivana Kuklová, Petra Pospíšilová, Lenka Krbková, Pavlína Koščová, Ivo Provazník, David Šmajs

**Affiliations:** 1 Department of Biomedical Engineering, Brno University of Technology, Brno, Czech Republic; 2 Department of Biology, Faculty of Medicine, Masaryk University, Brno, Czech Republic; 3 Department of Dermatology, 1st Faculty of Medicine, Charles University in Prague, Prague, Czech Republic; 4 Department of Children's Infectious Diseases, Faculty of Medicine and University Hospital, Masaryk University, Brno, Czech Republic; Instituto Butantan, BRAZIL

## Abstract

**Background:**

Pathogenic treponemes related to *Treponema pallidum* are both human (causing syphilis, yaws, bejel) and animal pathogens (infections of primates, venereal spirochetosis in rabbits). A set of 11 treponemal genome sequences including those of five *Treponema pallidum* ssp. *pallidum* (TPA) strains (Nichols, DAL-1, Mexico A, SS14, Chicago), four *T*. *p*. ssp. *pertenue* (TPE) strains (CDC-2, Gauthier, Samoa D, Fribourg-Blanc), one *T*. *p*. ssp. *endemicum* (TEN) strain (Bosnia A) and one strain (Cuniculi A) of *Treponema paraluisleporidarum* ecovar Cuniculus (TPeC) were tested for the presence of positively selected genes.

**Methodology/Principal findings:**

A total of 1068 orthologous genes annotated in all 11 genomes were tested for the presence of positively selected genes using both site and branch-site models with CODEML (PAML package). Subsequent analyses with sequences obtained from 62 treponemal draft genomes were used for the identification of positively selected amino acid positions. Synthetic biotinylated peptides were designed to cover positively selected protein regions and these peptides were tested for reactivity with the patient's syphilis sera. Altogether, 22 positively selected genes were identified in the TP genomes and TPA sets of positively selected genes differed from TPE genes. While genetic variability among TPA strains was predominantly present in a number of genetic loci, genetic variability within TPE and TEN strains was distributed more equally along the chromosome. Several syphilitic sera were shown to react with some peptides derived from the protein sequences evolving under positive selection.

**Conclusions/Significance:**

The syphilis-, yaws-, and bejel-causing strains differed relative to sets of positively selected genes. Most of the positively selected chromosomal loci were identified among the TPA treponemes. The local accumulation of genetic variability suggests that the diversification of TPA strains took place predominantly in a limited number of genomic regions compared to the more dispersed genetic diversity differentiating TPE and TEN strains. The identification of positively selected sites in *tpr* genes and genes encoding outer membrane proteins suggests their role during infection of human and animal hosts. The driving force for adaptive evolution at these loci thus appears to be the host immune response as supported by observed reactivity of syphilitic sera with some peptides derived from protein sequences showing adaptive evolution.

## Author summary

In the genus *Treponema* there are several human and animal pathogens that include the causative agent of syphilis (*Treponema pallidum* ssp. *pallidum*; TPA), the causative agent of yaws (*T*. *p*. ssp. *pertenue*; TPE), and the causative agent of endemic syphilis (*T*. *p*. ssp. *endemicum*; TEN). *T*. *paraluisleporidarum* causes venereal spirochetosis in rabbits. We used whole genome sequences of 11 treponemal strains together with additional 62 draft genomic data to identify genes evolving under positive selection. The identified genes evolving under positive selection partly overlapped with the genes previously reported as recombinant and were found to be different in treponemal subspecies. Since both genetic recombination and positive selection could allow a survival of pathogenic bacteria despite the human immune response, identification of such genes could predict the major antigens recognized by the human immune system and also identify the most suitable components for development of an anti-treponemal vaccine.

## Introduction

Adaptive evolution including positive selection plays crucial roles in the evolution of bacterial human pathogens and both have been well documented on a genome-wide scale in a number of bacterial genera including *Escherichia*, *Helicobacter*, *Neisseria*, *Listeria*, *Salmonella*, *Streptococcus*, *Campylobacter*, and *Actinobacillus* [1–8].

Pathogenic treponemes are both human and animal pathogens. Human pathogens include *Treponema pallidum* ssp. *pallidum* (TPA), the causative agent of syphilis, *T*. *p*. ssp. *pertenue* (TPE, the causative agent of yaws), and *T*. *p*. ssp. *endemicum* (TEN, the causative agent of bejel) while animal pathogens include TPE causing non-human primate infections [9–12], and *T*. *paraluisleporidarum* ecovar Cuniculus (TPeC; formerly denoted as *Treponema paraluiscuniculi*), the causative agent of venereal spirochetosis in rabbits, and *T*. *paraluisleporidarum* ecovar Lepus, which infects hares [13–15]. Although the recent work of Edmondson *et al*. [16] reported successful long-term cultivation of *T*. *pallidum* in a tissue culture system, most of the data on treponemal genetics comes from the whole genome sequencing studies [17,18].

The above-listed pathogens are monomorphic, i.e., highly similar at the genetic level and all the genomes of these pathogenic treponemes characterized to date share a genetic identity of 98% or higher [17,18]. The group of human pathogens (TPA, TPE, TEN) are even more related sharing a genetic identity over 99.7% [17,19,20]. Currently, 14 complete treponemal genomes have been published including six TPA genomes, six TPE genomes, one TEN genome, and one TPeC genome [12,19–29]. In addition, 23, 25, 8, and 6 draft whole genome sequences of treponemal strains or isolates were published recently by Arora *et al*. [30], Pinto *et al*. [31], Sun *et al*. [32], and Marks *et al*. [33], respectively.

The genomes of pathogenic treponemes related to *T*. *pallidum* contain no prophages or insertion sequence-elements [21,34], or plasmids [35]. Therefore, recombination is expected to be quite infrequent among these treponemes due to a lack of mobile genetic elements [21]. However, traces of both intragenomic DNA recombinations via gene conversion [36–38] and intergenomic homologous recombination after DNA horizontal gene transfer have been described [20,25]. In addition, traces of positive selection have been detected in previously published papers including TPA and TPE comparisons [19], comparisons within TPA strains [39], and detailed intrastrain analysis [40]. However, no comprehensive analysis of positively selected loci in the genomes of pathogenic treponemes has been performed to date.

Despite the fact that treponemes related to *Treponema pallidum* are monomorphic bacteria with extremely low level of genetic diversity [18], it has been shown that human immunity does not protect against different subspecies and not even against different syphilis strains [41]. Therefore, divergent genes encoding differences in proteomes of individual treponemal strains and subspecies are likely of importance for development of syphilis vaccine.

In this communication, the whole genome sequences of 11 treponemal strains were systematically analyzed for the presence of positive selection. The identified genes were further reanalyzed relative to all sequences available in 62 draft genomes published to date. The causative agents of syphilis, yaws, and bejel differed in sets of positively selected genes. Moreover, several synthetic peptides covering positively selected protein regions were found to interact with syphilitic sera.

## Materials and methods

### Strains used in this study

**Fig 1:**
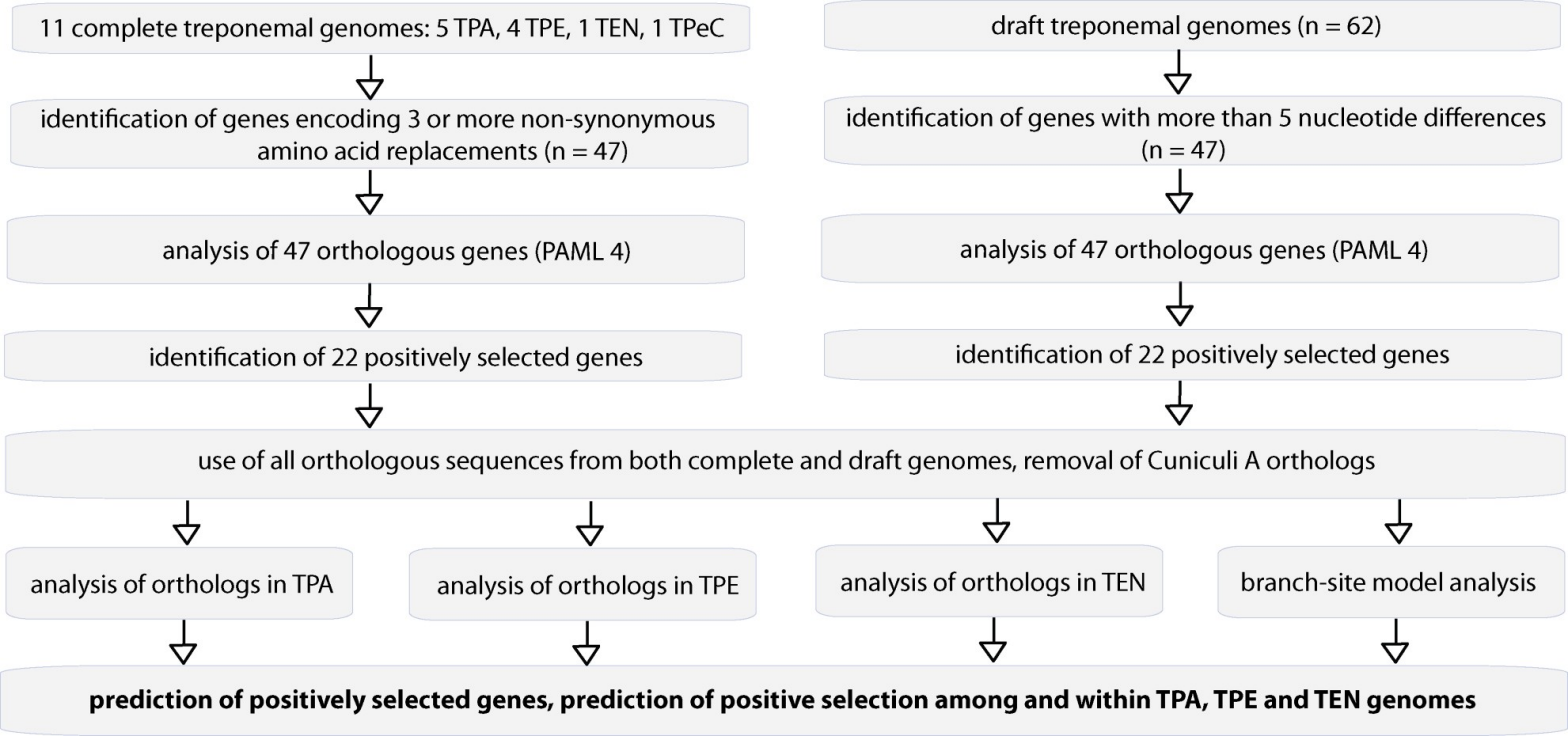
The algorithm used for identification of positively selected genes. The original search for positively selected genes started with identification of gene orthologs with 3 or more nucleotide differences leading to nonsynonymous amino acid replacements. The original search was performed on a set of 11 complete treponemal genomes listed in Table 1. Subsequently, orthologous gene sequences extracted from published treponemal draft genomes were used and the Cuniculi A orthologs were removed due to frequent sequential diversity and due to lack of pathogenicity of TPeC to humans. Orthologs from draft genomes were used when available and positively selected genes were analyzed within treponemal subspecies using branch-site PAML model analysis.

A set of 11 treponemal genomic sequences was examined in this study and included genomes of five TPA strains (Nichols, DAL-1, Mexico A, SS14, Chicago B), four TPE strains (CDC-2, Gauthier, Samoa D, Fribourg-Blanc), one TEN strain (Bosnia A), and one strain of TPeC (Cuniculi A). An overview of the complete genome sequences used is shown in Table 1. Subsequent analysis of selected genes was performed on additional 62 draft genomes (Arora *et al*. [30]; Pinto *et al*. [31]; Sun *et al*. [32]; GenBank genome TPA sequences [UW074B, UW189B, UW228B, UW254B, UW391B] and TEN Iraq B [CP032303.1]). The overall algorithm is shown in Fig 1.

**Table 1:** Treponemal genomes analyzed in this study.

TP strain*	Place and year of isolation	Reference	GenBank Accession number, Genome sequence reference
TPA Nichols	Washington, D.C., USA; 1912	[42]	CP004010.2, [21,26]
TPA DAL-1	Dallas, USA; 1991	[43]	CP003115.1, [29]
TPA SS14	Atlanta, USA; 1977	[44]	CP004011.1, [24,26]
TPA Mexico A	Mexico City, Mexico; 1953	[41]	CP003064.1, [25]
TPA Chicago	Chicago; 1951	[41]	CP001752, [22]
TPE CDC-2	Akorabo, Ghana; 1980	[45]	CP002375.1, [19]
TPE Gauthier	Brazzaville, Congo; 1960	[46]	CP002376.1, [19]
TPE Samoa D	Apia, Samoa; 1953	[41]	CP002374.1, [19]
TPE Fribourg-Blanc	Guinea; 1966	[9,10]	CP003902.1, [12]
TEN Bosnia A	Bosnia; 1950	[47]	CP007548, [20]
TPeC Cuniculi A	unknown; before 1957	[41]	CP002103.1, [27]

*Additional genome sequences of TPE Ghana-051 and CDC 2575 became available recently [28] and TPA Sea81-4 was published as a whole genome sequence [23].

### Identification of genes under adaptive evolution

The TPE Samoa D was used as a reference genome, and all 1068 orthologous genes were extracted from the other 10 complete genomes using given annotation coordinates. The orthologous sequences from the complete genomes were aligned at the codon level using Matlab R2013a software and the Bioinformatics Toolbox. Only genes where at least three nonsynonymous mutations at different sites occurred were further analyzed with respect to the presence of positive selection. A BLAST search was used to determine orthologous sequences in the draft genomes. These sequences were aligned in Matlab at the nucleotide level because large number of ambiguous sites precluded proper automatic ORF localization. Then the sequences from the draft genomes were scanned for nucleotide differences. After filtering sites with unknown nucleotides, insertions, and deletions, only orthologs with more than five nucleotide differences at different sites were analyzed further. These orthologs were aligned with the corresponding genes from the complete genomes to determine the ORF and identify the number of nonsynonymous mutations. At the same time, the TPeC orthologs were excluded in this step due to frequent sequential diversity and due to the lack of pathogenicity of TPeC to humans. The removal of the TPeC orthologs did not change the number of detected positively selected genes. Compared to an analysis of whole genomes, no new locus with more than three nonsynonymous mutations at different sites was identified during the analysis of draft genomes.

For each analyzed gene from the complete genomes, a maximum likelihood phylogenetic tree with 50 bootstrap replicates was constructed using MEGA 6 [48]. Different evolution models (Kimura 2-parameter [49], Tamura 3-parameter [50], and Tamura-Nei [51]) were applied to each gene. The trees of each gene were compared by calculating Robinson-Foulds distances [52] using R software (packages phytools and phangorn) [53]. The comparison showed that the choice of the evolution model did not significantly change the topology of the tree; the Tamura-Nei evolution model was chosen for the construction of all phylogenetic trees.

A calculation of mutational rate ratio ω between two gene sequences was the basis for the positive selection analysis. The ω was calculated as a ratio of nonsynonymous to synonymous mutational rates. The ratio indicates negative purifying selection (0 < ω < 1), neutral evolution (ω = 1), and positive selection (ω > 1) [54]. A set of selected genes from complete genomes was tested relative to positive selection using the maximum likelihood method using the CODEML of the PAML software package [55]. PAML version 4 [56] and its user interface PAMLX [57] were used in our study. For each analyzed gene, its maximum likelihood phylogenetic tree was used as an input tree. The CODEML offers several different codon evolutionary models, and the statistical likelihood ratio test (LRT) was used to compare the codon evolutionary model to the null model. The Bayes empirical Bayes method (BEB) [58] was then used to evaluate the posterior probability of sites considered to have been positively selected.

The CODEML models could produce different results (i.e., a list of sites under positive selection) since they calculate different parameter estimates. Site models allow ω to vary in each site (codon) within the gene. Statistical testing was required for sites with ω > 1. Two pairs of models were predominantly used since their LRTs have low false-positive rates. M1a (nearly neutral evolution) was compared to M2a (positive selection) [58,59] and M7 (beta) was compared to M8 (beta & ω) [60]. Our preliminary testing found that the two model pairs gave the same or very similar results. Therefore we chose to use the M7-M8 model pair. The M7 model is a null model that allows 10 classes of sites with a ω beta-distribution within the interval 0 ≤ ω ≤ 1. Sites with ω > 1 are not allowed. The alternative M8 model adds an eleventh class of sites with ω > 1. Each site was tested to determine the class to which it belongs. The LRT compares twice the log-likelihood difference 2Δ*l* = 2(*l*_1_-*l*_0_) between the M7 model (log likelihood value *l*_0_) and the M8 model (log likelihood value *l*_1_) to the χ^2^ distribution [61]. If the twice log-likelihood difference is above a critical χ^2^ value, then the null model is rejected, and the positive selection is statistically significant.

A considerable disadvantage of the site models is that ω was calculated as an average over all codons of the site. Therefore, the site models are not suitable for the data where ω also varies between lineages. In contrast, the branch-site models search for positive selection in sites and pre-specifies lineages where different rates of ω may occur [62]. Sequences of lineages are *a priori* divided into a group of foreground lineages where positive selection may occur and group of background lineages where only purifying selection or neutral evolution occurs. We used branch-site model A, which allows four classes of sites and different setups of foreground lineages to be tested depending on the gene phylogeny. In branch-site model A, all lineages under purifying selection with a low value of ω_0_ belong to site class 0. Weak purifying selection and neutral evolution with ω_1_ near to value 1 are allowed in site class 1. In site class 2a, a proportion of class 0 sites in foreground lineages is under positive selection with ω_2_ > 1. Similarly, site class 2b is a proportion of class 1 sites under positive selection with ω_2_ > 1. The null model for LRT has ω_2_ = 1. Critical values of LRT (2Δl) are 2.71 at 5% and 5.41 at 1% [63]. The posterior probabilities of suggested sites under positive selection were calculated using the BEB method.

The average pairwise p-distances (APD) and average number of mutations (transitions and transversions), calculated using MEGA-X [64], were used to evaluate genetic diversity. A pairwise deletion of sites with gaps/missing data was used. The Fisher exact statistical test was used to assess the significance of the changes between average numbers of mutations.

### Synthetic peptides and chemiluminescent detection of serological reactions

Synthetic biotinylated peptides, covering protein regions containing positively selected residues, were designed. Peptide synthesis was performed by JPT Peptide Technologies (Berlin, Germany) on a 50–200 nmol scale. The lyophilized peptides were resuspended in TBS buffer (25 mM Tris, 150 mM NaCl, pH = 7.2) at 1 mM concentrations and were stored at −20°C. Prior to further use, synthetic peptides were diluted 1000x in TBS buffer.

Streptavidin-coated 96 well plates (Pierce Streptavidin Coated High Binding Capacity White 96-Well Plates; Thermo Scientific, Rockford, USA) were washed three times with 200 μl of washing buffer (TBS buffer containing Tween 20 (0.05%) and Bovine Serum Albumin, BSA (0.1%) (Sigma-Aldrich, Prague, Czech Republic); then 100 μl of diluted peptide was added to each well and incubated for 30 min at room temperature as recommended by the manufacturer (Thermo Scientific) with mild shaking. Subsequently, each well was washed three times with washing buffer (200 μl in each step); then 100 μl of blocking buffer SuperBlock Blocking Buffer in TBS (Thermo Scientific) were added and incubated for 30 min at room temperature with mild shaking. Each well was washed three times with washing buffer (200 μl in each step) and 100 μl of diluted sera (1:500 in washing buffer) were added and incubated for 30 min at room temperature with mild shaking. Each well was washed three times with washing buffer (200 μl in each step) and 100 μl of diluted secondary antibody conjugated with horseradish peroxidase were subsequently added (1:2000 in washing buffer; Goat Anti-Human IgG/IgA/IgM Horseradish Peroxidase Conjugate; Life Technologies, Carlsbad, USA) and incubated for 30 min at room temperature with mild shaking. Each well was then washed three times with washing buffer (200 μl in each step); then 100 μl of chemiluminescent detection solution (Super Signal ELISA Pico Chemiluminescent Substrate; Thermo Scientific) was added. Luminescence was measured on a TriStar^2^ LB 942 luminometer with a Modular Multimode Microplate Reader (Berthold Technologies, Bad Wildbad, Germany). Each experiment was performed at least three times. A signal was considered positive when it was higher than the average of the three lowest values for each serum plus five standard deviations of the average value.

### Ethics statement

The human sera were collected from adult patients diagnosed with syphilis at the Department of Dermatology, 1st Faculty of Medicine, Charles University, Prague, Czech Republic. Sera from child patients diagnosed with Lyme disease were obtained from the Department of Children's Infectious Diseases, Faculty of Medicine and University Hospital, Masaryk University, Brno, Czech Republic. All clinical samples were obtained after patients or parents of involved children signed an informed consent. The design of the study was approved by the ethics committee of the Faculty of Medicine, Masaryk University. All human sera were collected under established guidelines.

## Results

### Identification of positively selected genes

A comparison of 47 orthologous gene sequences from the complete genomes where at least three nonsynonymous mutations at different sites occurred was used to identify positively selected genes using the site and branch-site models of the CODEML in PAML package [55]. The completely sequenced genomes are listed in Table 1 and include 11 genomes. In addition, 25 draft TPA genomes [31], 23 draft TPA and TPE genomes [30], 8 TPA genomes [32], 5 TPA genomes from GenBank (UW074B, UW189B, UW228B, UW254B, UW391B), and one TEN (Iraq B) were also analyzed. The overall algorithm is shown in Fig 1.

In all cases of complete treponemal genomes, the genome structure was identical or very similar allowing straightforward identification of gene orthologs. However, in many cases, draft genome sequences were either incomplete or contained many ambiguous bases precluding their use in analyses. This resulted in a variable number of sequences used for identification of positively selected sites within individual loci. Altogether, 22 positively selected genes were identified in the TP genomes using site model analysis in PAML (Table 2). The number of positively selected amino acid sites varied from 1 to 65, with a median value of 8.5. A list of positively selected protein sites identified using PAML software (site and branch-site models) as well as PAML-identified positively selected protein sites within treponemal subspecies are shown in S1 Table.

**Table 2:** A set of 22 genes evolving under adaptive evolution that was identified using site and branch-site model analysis in the PAML program.

Gene	Gene name	Protein	No. of positively selected protein sites identified by PAML site or branch-site model (no. of analyzed sequences)	Previously published evidence of recombination	References	Gene average pairwise p-distances
**TP0117**	*tprC*	Tpr protein C	22 (41)	+	[38]	0.009171
TP0126b		hypothetical protein	2 (69)	-		0.005899
**TP0131**	*tprD*	Tpr protein D	65 (41)	+	[38]	0.033553
**TP0133**		outer membrane protein^a^	3 (66)	+	[20]	0.004718
**TP0136**		fibronectin binding protein	5 (54)	**+**	[65]	0.016599
TP0314		subtilisin-like protein^a^	7 (49)	-		0.026378
TP0316	*tprF*	Tpr protein F	5 (33)	-		0.003250
**TP0317**	*tprG*	Tpr protein G	8 (39)	+	[38]	0.003241
**TP0326**	*bamA*	BamA	7 (64)	+	[20,25,66]	0.002131
TP0462		lipoprotein, subtilisin-like protein^a^	44 (60)	-		0.009186
**TP0488**	*mcp2*	methyl-accepting chemotaxis protein	50 (64)	+	[20,25,67]	0.002691
TP0515		outer membrane protein	15 (66)	-		0.001381
**TP0548**		FadL-like protein^b^	24 (52)	+	[67]	0.010881
TP0619		Fe, Mn superoxide dismutase^a^	7 (40)	-		0.029166
**TP0620**	*tprI*	Tpr protein I	14 (38)	+	[38]	0.006651
**TP0621**	*tprJ*	Tpr protein J	52 (40)	+	[38]	0.023909
TP0733		OprG/OmpW-like ion-channel^b^	1 (62)	-		0.005125
**TP0856**		FadL-like protein^b^	16 (62)	+	[68]	0.003663
**TP0858**		FadL-like protein^b^	2 (61)	+	[33,68]	0.005894
TP0859		FadL-like protein^b^	7 (33)	-		0.004191
**TP0865**		FadL-like protein^b^	13 (64)	+	[30]	0.004340
**TP1031**	*tprL*	Tpr protein L	9 (58)	+	[20]	0.007283

^a^protein predictions by Naqvi *et al*. [69]

^b^protein predictions by Radolf and Kumar [70]

Average pairwise p-distances (APD) were calculated for each of the 22 genes from 10 complete genomes (Cuniculi A genome was removed from analysis) and from the draft genomes. The APD value for each gene was compared with APD_w10_ = 0.000525 of the 10 complete genomes without 54 variable loci (listed in S2 Table) which can be considered as a background level of polymorphism. All 22 genes evolving under adaptive evolution had elevated nucleotide substitution density.

Out of these 22 genes, 14 genes were previously reported as recombinant (Table 2). These genes are listed in Table 3. Functionally, these genes comprised the *tpr* genes (*tprC*, *D*, *G*, *I*, *J*, *L*), outer membrane proteins (TP0133, TP0136, TP0548, TP0856, TP0858, TP0865), and genes encoding the outer membrane biogenesis protein (TP0326 [BamA]) and methyl-accepting chemotaxis protein (TP0488 [Mcp-2]). Putative recombination loci were most frequently identified in TEN strain (n = 9) while in other treponemes there were fewer predicted recombinant loci (TPA, n = 7; TPeC, n = 1; TPE, n = 1). When recombinant sequences were removed from analyses, positive selection among 14 these loci (Table 3) was found mostly within TPA strains or isolates (n = 9), within TPE (n = 4), between TEN and TPA/TPE sequences (n = 2), between TPA and TPE strains (n = 1) and between TPA and TPE/TEN sequences (n = 1).

**Table 3:** A set of 14 positively selected genes with previously detected recombination events.

Gene	Gene name	Protein	Putative recombination in	Evidence of positive selection among non-recombinant sequences
TP0117	*tprC*	Tpr protein C	TPA, TEN	within TPA between TPA and TPE
TP0131	*tprD*	Tpr protein D	two alternative *tprD* and *tprD2* alleles^a^	no
TP0133		outer membrane protein^b^	TEN	between TEN/TPE and TPA
TP0136		fibronectin binding protein	TPA	within TPA
TP0317	*tprG*	Tpr protein G	TPA	within TPA
TP0326	*bamA*	BamA	TPA, TEN	within TPA
TP0488	*mcp2*	methyl-accepting chemotaxis protein	TPA, TEN	within TPA
TP0548		FadL-like protein^c^	TEN	within TPA within TPE
TP0620	*tprI*	Tpr protein I	TPE	within TPE
TP0621	*tprJ*	Tpr protein J	TPA, TPeC	within TPA
TP0856		FadL-like protein^c^	TEN	between TEN and TPA/TPE
TP0858		FadL-like protein^c^	TEN	within TPE
TP0865		FadL-like protein^c^	TPA, TEN	within TPA within TPE between TEN and TPA/TPE
TP1031	*tprL*	Tpr protein L	TEN	within TPA

^a^*tprD* and *tprD2* alleles existed in both TPA and TPE strains [18,71]

^b^protein predictions by Naqvi *et al*. [69]

^c^protein predictions by Radolf and Kumar [70]

### Positively selected genes with no recombination events described so far

A list of the 8 positively selected genes is shown in Table 4. These genes include *tprF* gene, genes encoding outer membrane proteins (TP0515, TP0733, TP0859), subtilisin-like proteins (TP0314, TP0462), enzymes (TP0619), and hypothetical protein (TP0126b). Evidence of positive selection from analyses performed within the strains belonging to different treponemal subspecies and analyses from the PAML branch-site model revealed that positive selection was found mostly between TPA and TPE strains (n = 6), and within TPA strains or isolates (n = 2). In one case (TP0859), positive selection was found both between TPA and TPE strains (n = 1), and between TEN and TPA/TPE (n = 1).

**Table 4:** Positively selected genes revealed by the PAML program with no recombination events described so far.

Gene	Gene name	Protein	Positively selected branch
TP0126b		hypothetical protein	between TPA and TPE
TP0314		subtilisin-like protein^a^	between TPA and TPE
TP0316	*tprF*	Tpr protein F	between TPA and TPE
TP0462		lipoprotein, subtilisin-like protein^a^	within TPA
TP0515		outer membrane protein	within TPA
TP0619		Fe, Mn superoxide dismutase^a^	between TPA and TPE
TP0733		OprG/OmpW-like ion channel^b^	between TPA and TPE
TP0859		FadL-like protein^b^	between TPA and TPE between TEN and TPA/TPE

^a^protein predictions by Naqvi *et al*. [69]

^b^protein predictions by Radolf and Kumar [70]

**Fig 2:**
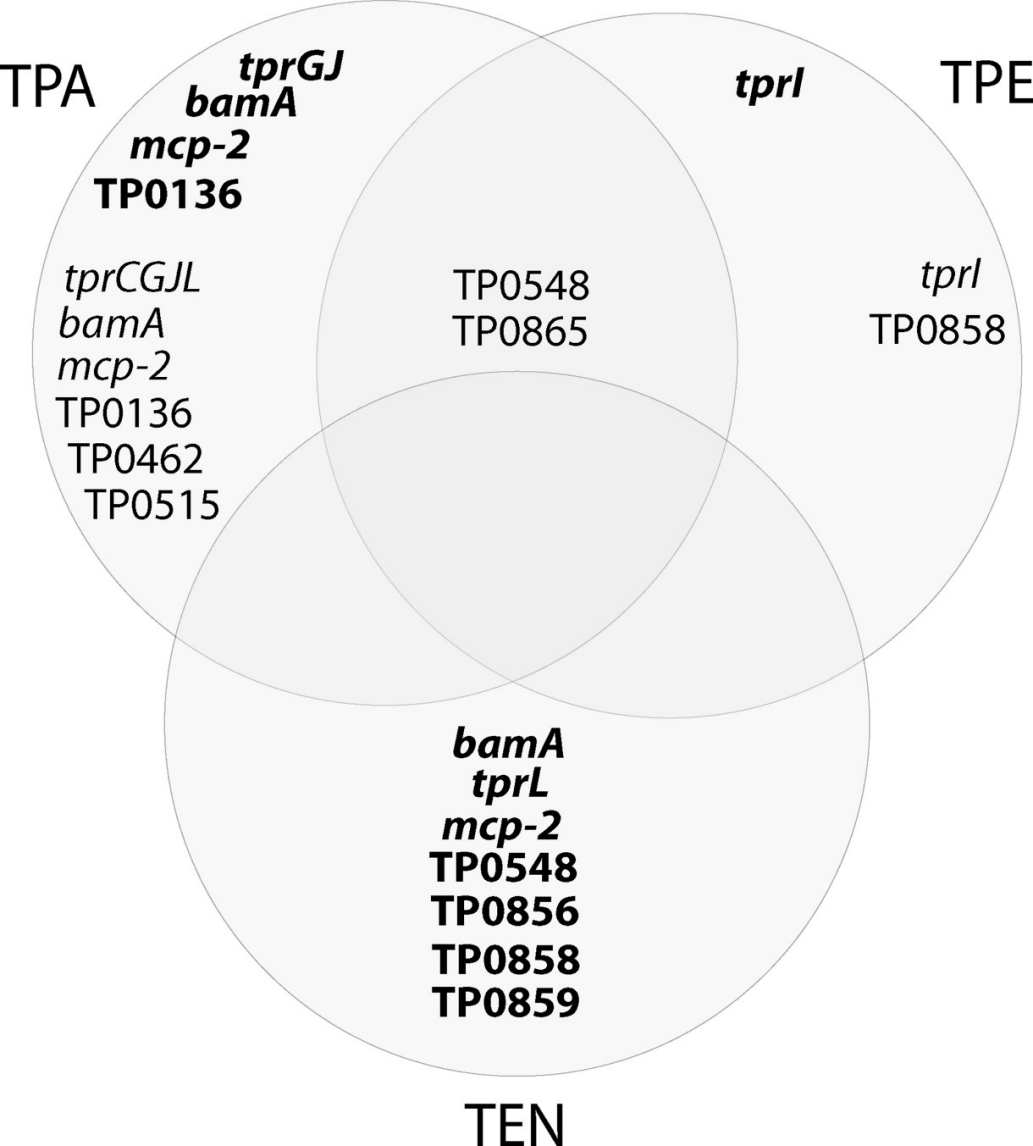
Positively selected genes as well as positively selected genes with previously identified recombination event that were identified within particular subspecies. Genes identified as recombinant in a particular treponemal subspecies are shown in bold. Positively selected genes with no evidence of recombination are shown in regular version. Positively selected genes identified between subspecies of treponemes, but not within any of them, are not shown. Note that positively selected genes occur mostly within the TPA and the recombinant genes are within the TEN genomes. The TP0548 and TP0865 genes were found to be positively selected within TPA and also within TPE subspecies.

Identified positively selected genes in various treponemal species and subspecies are shown in Table 5 and Fig 2. While Table 5 lists all identified recombinant and positively selected genes in TPA, TPE, and TEN groups of strains or isolates, Fig 2 shows only recombinant or positively selected genes that were identified within a particular subspecies.

**Table 5:** Positively selected genes and the corresponding proteins in different treponemal species and subspecies. Proteins previously reported as recombinant out of the positively selected proteins are also shown.

*Treponema pallidum* subspecies	Recombinant proteins	Positively selected proteins
**TPA**	TprC, G, J, BamA, Mcp-2, TP0136	TprC, F, G, J, L, BamA, Mcp-2 TP0126b, TP0133, TP0136, TP0314, TP0462, TP0515, TP0548, TP0619, TP0733, TP0859, TP0865
**TPE**	TprI	TprE, F, I TP0126b, TP0133, TP0314, TP0548, TP0619, TP0733, TP0858, TP0859, TP0865
**TEN**	BamA, Mcp-2, TprL TP0548, TP0856, TP0858	TprL TP0856, TP0858, TP0865

### Distribution of genetic diversity among TPA, TPE, and TEN strains

To test whether genetic diversity in treponemal genomes was distributed evenly along the chromosome, an average pairwise p-distance (APD) and average number of mutations (ANM, transitions and transversions) were calculated (Table 6) for concatenation of 54 genes (S2 Table) representing a total length of 86.8 kbp (7.6% of the genome length). These 54 genes included the *tpr* genes, genes with traces of possible recombination events (according to Gray *et al*. [38], and Čejková *et al*. [19]), genes showing inter-strain variability between TPE and TPA strains and their paralogs (according to Čejková *et al*. [19]), genes showing intra-strain variability [40], and previously identified positively selected genes (according to Čejková *et al*. [19]) (S2 Table). The TPA strains contained greater genetic diversity within these genes compared to TPE strains (APD_(TPA)_ = 420.0*10^−5^ versus APD_(TPE)_ = 384.2*10^−5^). Note that the average pairwise p-distances within TPA strains (compared to TPE/TEN strains) are lower when complete genomes were analyzed but higher within selected 54 loci (Table 6). In addition, genetic distance within TPA strains (compared to TPE/TEN strains) is markedly lower in complete genomes without selected 54 genes compared to whole genome analyses (p = 0.0008).

**Table 6:** Average pairwise p-distances (APD) and average number of mutations (ANM, transitions + transversions) within TPA, within TPE/TEN, and between TPA and TPE/TEN, for whole complete genomes, for selected 54 genomic loci, and for complete genomes without selected 54 loci.

	Whole genomes APD (ANM)		54 selected loci (S2 Table) APD (ANM)		Genomes without 54 loci APD (ANM)	
**TP subspecies**	Within Group	Between groups	Within group	Between groups	Within group	Between Groups
TPA	0.000415^a^ (472.7)	TPA-TPE/TEN 0.001908 (2171.4)	0.004200 (361.8)	TPA-TPE/TEN 0.015227 (1297.0)	0.000105^a^ (110.9)	TPA-TPE/TEN 0.000831 (874.4)
TPE/TEN	0.000455^a^ (517.4)		0.003842 (325.7)		0.000182^a^ (191.7)	

^a^Statistically significant difference for genetic distance within TPA strains compared to TPE/TEN strains when complete genomes and genomes without selected 54 genes were compared (The Fisher exact test, p = 0.0008).

### Serological reactivity of human syphilis and Lyme disease sera with synthetic biotinylated peptides derived from positively selected proteins

Synthetic biotinylated peptides were designed to cover protein regions where positively selected amino acid residues were detected. These peptides were tested for reactivity with the patient's syphilis sera (Table 7). As a control, serum from a patient with Lyme disease was used. A positive result was obtained for one to seven peptides (out of 8 tested) depending on the serum used. At the same time, serum obtained from Lyme disease patient failed to recognize peptides derived from treponemal proteins but did react with peptides derived from borrelial protein ErpA.

**Table 7:** Serological reactivity of a patient's syphilis and Lyme disease sera recognizing synthetic peptides corresponding to protein regions containing positively selected amino acid residues. Peptides were designed to cover protein regions containing several positively selected amino acid positions.

Peptide	Derived from gene	Protein function	Sequence	Syphilis sera			Lyme disease serum
				350^a^	356^b^	405^c^	B0403201^d^
TPA51S	TP0117	TprC	YVFYRNNGGYELNRVVPSGI	+ ^e^			
TPE83	TP0136	fibronectin binding protein	GNSANGGGGGGGCGS	+			
TPA04	TP0314	subtilisin-like protein	LQPSSSSYSAGNWHR	+	+	+	
TPA58	TP0316	TprF	HQSNADADCRLPATG	+		+	
TPA61-S	TP0462	lipoprotein, subtilisin-like protein	TPSTVLDKTNGAIR	+		+	
TPA64-S	TP0515	outer membrane protein	YRLHSEPPSSGSRQ	+			
TPA17	TP0619	Fe, Mn superoxide dismutase	LGQGLLQPSSSSYSA				
TPA21	TP0733	OprG/OmpW-like ion channel	GDIASSPDKCRAVGL	+		+	
BB-ErpA-Bval	*erpA*	ErpA	KIKNKDTNSSWIDL				+

^a^Human serum comes from a 33-year-old patient that was syphilis positive for VLDR (1:4), TPHA and western blot IgG test.

^b^Human serum comes from a 32-year-old patient that was syphilis positive for VLDR (1:8), TPHA and western blot IgG test.

^c^Human serum comes from a 21-year-old patient that was syphilis positive for VLDR (1:128), TPHA and western blot IgG and IgM test.

^d^Human serum comes from a 12-year-old patient with Lyme disease positive for ELISA IgG and western blot IgM and IgG tests.

^e^+, signal above threshold; for each peptide, an average of the three lowest values (out of 9) plus 5 standard deviations was used as a threshold.

## Discussion

In this study, 22 genes showing traces of positive selection were identified among the TPA, TPE, and TEN genomes. Within this group of genes, recombination was previously reported in 14 genes. Genes with previously detected recombination events were often found to contain positively selected amino acid residues both among the recombinant and the non-recombinant sequences, which indicates that both recombination and positive selection are different mechanisms of treponemal adaptive molecular evolution. Adaptive evolution is common to many bacterial pathogens and can usually be found in genes important to the interaction between the host and the pathogen, i.e., where new protein variants are of selective advantage for the survival of the pathogenic strain. The immune pressure from the host favors, in microbial genes encoding proteins exposed on the surface of the pathogen, emerging genetic variants, which due to immune evasion, get positively selected. In *Escherichia coli*, positive selection is limited to a few dozen genes [3] while in several other genomes, including *Streptococcus* [1] and *Campylobacter* [2], traces of positive selection have been found in more than half of the core genome.

The extent to which adaptive evolution of different bacterial pathogens differs depends on several bacterial features including bacterial mutation rate, frequency of genetic recombination and horizontal gene transfer, and genome size. Genetic recombination occurs more frequently in *Neisseria* [8] and *Helicobacter* [5] compared to several bacterial pathogens such as *Escherichia* [3], *Salmonella* [4], and *Listeria* [6]. Moreover, compared to *E*. *coli*, *Helicobacter pylori* has about a 100-times higher mutation rate due to the lack of a highly efficient DNA repair system [72]. Treponemes related to *T*. *pallidum* represent bacterial pathogens with small genomes, with an extreme paucity of outer membrane proteins [73], high genetic similarity, and a relatively low mutation rate [28]. Moreover, there are no known mechanisms of horizontal gene transfer in syphilis, bejel, and yaws treponemes [18]. These features of pathogenic treponemes are consistent with a relatively small number of positively selected genetic loci, which consists of just 22 genes (2.1% of all protein-encoding genes). Moreover, this situation also partly reflects the fact that the number of determined treponemal genomes is quite low due to the difficulties in long-term cultivation of treponemes [16]; the sequenced genomes currently available do not reveal the entire genetic variability present among human pathogenic treponemes. A recently developed MLST typing of TPA treponemes [74,75] revealed a number of genetic variants of the TP0705 gene and the vast majority of these variants resulted in amino acid replacements, which suggests positive evolution at this locus. This locus was not identified in this study because of the limited variability present in the currently available genome sequences. It is therefore expected that the list of positively selected/recombinant treponemal loci will grow larger as the number of additional genomes accumulate.

Among the genes identified in this study, a substantial number of genes were shown to have evidence of recombination event. Several recent studies revealed that genetic recombination in pathogenic treponemes is not only limited to intra-genomic homologous recombination and gene conversion events involving rDNA loci, *tpr* genes and their paralogs, and the TP0856 and TP0858 genes [36,38,68]. In addition to intra-genome recombinations, two genes, TP0326 and TP0488, in the TPA Mexico A genome, were found as a result of recombination with exogenous DNA, likely as a result of DNA uptake and chromosome incorporation during coinfection with a different treponemal subspecies [25]. Similar recombinations were detected in a TPA isolate from South Africa [66]. In the work of Grange *et al*. [76], TEN strain 11qj within the TP0548 locus and a nucleotide sequence almost identical to TPE strains [67,77,78] indicating that the TP0548 locus in other TEN strains is also a result of an interstrain recombination event. In the genome of TPA Sea84-1, the TP0621 locus revealed sequences identical to TPE [23]. Moreover, whole-genome sequencing of TEN Bosnia A revealed several genomic loci similar to TPA strains [20]. All these findings suggest that genetic recombinations of exogenous DNA into the chromosomal loci of treponemes are rare but detectable events. In addition, these findings suggest that the corresponding recombination could be of selective advantage during host infection given the fact that the recombination of foreign DNA without available horizontal gene transfer mechanisms is quite infrequent.

In the work of Arora *et al*. [30], the authors find the genes with predicted putative recombinant regions (e.g., TP0136, TP0462, TP0548, TP0733, TP0894-898) using a phylogenetic incongruence method, ClonalFrameML, and Gubbins, overlapping with genes identified in our study. However, several predicted recombinant genes comprising TP0179, TP0313, TP0315, TP0967, TP0968 [30], were not identified in this study reflecting the fact that not all recombination events result in detectable positive selection signal.

Out of the 22 genes showing adaptive evolution identified in this study, nine genes (40.9%) were identified in the set of 71 genes showing intra-strain heterogeneity (reviewed in Šmajs *et al*. [18]). Since most of the observed intra-strain heterogeneity resulted in non-synonymous amino acid changes, identified intra-strain heterogeneity should be considered as ongoing adaptive evolution and, as such, it is not surprising that both sets of positively selected/recombinant genes and genes showing intra-strain heterogeneity overlapped considerably.

Functionally, the genes showing adaptive evolution identified in this study were the *tpr* genes (*tprC*, *D*, *F*, *G*, *I*, *J*, *L*), outer membrane proteins (TP0133, TP0136, TP0515, TP0548, TP0733, TP0859, TP0865) and genes encoding outer membrane biogenesis protein (BamA), lipoproteins (TP0462, TP0856, TP0858), methyl-accepting chemotaxis protein (TP0488), and three other proteins (TP0126a, TP0314, TP0619). The driving force for adaptive evolution at these loci thus appears to be the host immune response. The observed reactivity of syphilitic sera with some peptides derived from protein sequences evolving under positive evolution supports this prediction.

This study has several limitations. First, the number of analyzed genomes was quite limited, especially regarding the TEN genomes. In addition, in recently published draft genomes of TPA, the candidate genes are often only partially sequenced. With a much larger set of treponemal sequences, one can expect a higher number of positively selected genes. The second major limitation was the disproportionality in the number of analyzed genes per species/subspecies, which reflects genome availability. Another limitation is related to the fact that the analyzed complete genomes were obtained from treponemal strains propagated in rabbits and could therefore reflect adaptation of treponemes to this host. However, the analysis of draft genome sequences in this study obtained directly from clinical material suggests that the observed traces of positive selections are present also during infection of humans. Moreover, the identified positively selected positions may represent recent mutations that were not yet removed by negative selection.

In this study, a detailed analysis of traces of positive selection in 3 *T*. *pallidum* subspecies including ssp. *pallidum* (TPA), ssp. *pertenue* (TPE), and ssp. *endemicum* (TEN) enabled us to classify most of the identified positively selected genes to a particular subspecies when analyses were performed separately within strains and isolates of the same subspecies or when a PAML branch-site model was used to identify lineages with positively selected loci. The majority of positively selected genes were identified within the TPA and TPE genomes, likely as a result of the highest number of available sequences for these subspecies. However, TPA sets of positively selected genes differed from TPE genes. Among TPA, members of the paralogous *tpr* family (*tprCGJ*) and the TP0136 paralogous gene family (TP0136, TP0462) prevailed, while among TPE, a paralogous gene family containing TP0856, TP0858, TP0859, TP0865, showed adaptive evolution. Interestingly, the genes belonging to the latter family (TP0548, TP0856, TP0858, TP0859) were found to be recombinant among TEN genomes. These findings suggest that genomic loci showing signs of adaptive evolution could differ between TPA and TPE/TEN strains/isolates. This finding supports the observed and consistent genetic differences between treponemal subspecies TPA, TPE, and TEN, and shows that the ways TPA and TPE strains interact with a host during infection is different. Although some authors suggest that the subspecies classification is a case of opportunity and not the consequence of genetic and biological differences [79,80], our findings support the latter explanation.

## Supporting information

S1 Table A list of positively selected protein positions identified by PAML software using site and branch-site models and PAML analysis within treponemal subspecies. (XLSX)

S2 Table A set of 54 genes representing a total length of 86.8 kbp (7.6% of the genome length) that was removed from the whole genome sequences. (XLSX)

## References

[1] Lefébure T, Stanhope MJ. Evolution of the core and pan-genome of *Streptococcus*: positive selection, recombination, and genome composition. Genome Biol. 2007;8: R71 10.1186/gb-2007-8-5-r71 17475002 PMC1929146

[2] Lefébure T, Stanhope MJ. Pervasive, genome-wide positive selection leading to functional divergence in the bacterial genus *Campylobacter*. Genome Res. 2009;19: 1224–1232. 10.1101/gr.089250.108 19304960 PMC2704436

[3] Petersen L, Bollback JP, Dimmic M, Hubisz M, Nielsen R. 2007. Genes under positive selection in *Escherichia coli*. Genome Res. 2007;17: 1336–1343. 10.1101/gr.6254707 17675366 PMC1950902

[4] Soyer Y, Orsi RH, Rodriguez-Rivera LD, Sun Q, Wiedmann M. Genome wide evolutionary analyses reveal serotype specific patterns of positive selection in selected *Salmonella serotypes*. BMC Evol Biol. 2009;9: 264 10.1186/1471-2148-9-264 19912661 PMC2784778

[5] Suerbaum S, Smith JM, Bapumia K, Morelli G, Smith NH, Kunstmann E, et al Free recombination within *Helicobacter pylori*. Proc Natl Acad Sci USA. 1998;95: 12619–12624. 10.1073/pnas.95.21.12619 9770535 PMC22880

[6] Tsai YH, Maron SB, McGann P, Nightingale KK, Wiedmann M, Orsi RH. Recombination and positive selection contributed to the evolution of *Listeria monocytogenes* lineages III and IV, two distinct and well supported uncommon *L*. *monocytogenes* lineages. Infect Genet Evol. 2011;11: 1881–1890. 10.1016/j.meegid.2011.08.001 21854875 PMC3224679

[7] Xu Z, Chen H, Zhou R. Genome-wide evidence for positive selection and recombination in *Actinobacillus pleuropneumoniae*. BMC Evol Biol. 2011;11: 203 10.1186/1471-2148-11-203 21749728 PMC3146884

[8] Yu D, Jin Y, Yin Z, Ren H, Zhou W, Liang L, et al A genome-wide identification of genes undergoing recombination and positive selection in *Neisseria*. Biomed Res Int. 2014;2014: 815672 10.1155/2014/815672 25180194 PMC4142384

[9] Fribourg-Blanc A, Mollaret HH, Niel G. Serologic and microscopic confirmation of treponemosis in Guinea baboons. Bull Soc Pathol Exot Filiales. 1966;59: 54–59. 5333741

[10] Fribourg-Blanc A, Mollaret HH. Natural treponematosis of the African primate. Primates Med. 1969;3: 113–121. 5006024

[11] Knauf S, Batamuzi EK, Mlengeya T, Kilewo M, Lejora IA, Nordhoff M, et al Treponema infection associated with genital ulceration in wild baboons. Vet Pathol. 2012;49: 292–303. 10.1177/0300985811402839 21411621

[12] Zobaníková M, Strouhal M, Mikalová L, Čejková D, Ambrožová L, Pospíšilová P, et al Whole genome sequence of the *Treponema* Fribourg-Blanc: unspecified simian isolate is highly similar to the yaws subspecies. PLoS Negl Trop Dis. 2013;7: e2172 10.1371/journal.pntd.0002172 23638193 PMC3630124

[13] Jacobsthal E. Untersuchungen uber eine syphilisahnliche Spontanerkrankungen des Kaninchens (*Paralues cuniculi*). Derm Wschr. 1920;71: 569–571.

[14] Smith JL, Pesetsky BR. The current status of *Treponema cuniculi*. Review of the literature. Br J Vener Dis. 1967;43: 117–127. 10.1136/sti.43.2.117 5338028 PMC1047863

[15] Lumeij JT, Mikalová L, Šmajs D. Is there a difference between hare syphilis and rabbit syphilis? Cross infection experiments between rabbits and hares. Vet Microbiol. 2013;164: 190–194. 10.1016/j.vetmic.2013.02.001 23473645

[16] Edmondson DG, Hu B, Norris SJ. Long-Term In Vitro Culture of the Syphilis Spirochete *Treponema pallidum subsp*. *pallidum*. MBio 2018;9: e01153–18. 10.1128/mBio.01153-18 29946052 PMC6020297

[17] Šmajs D, Norris SJ, Weinstock GM. Genetic diversity in *Treponema pallidum*: implications for pathogenesis, evolution and molecular diagnostics of syphilis and yaws. Infect Genet Evol. 2012;12: 191–202. 10.1016/j.meegid.2011.12.001 22198325 PMC3786143

[18] Šmajs D, Strouhal M, Knauf S. Genetics of human and animal uncultivable treponemal pathogens. Infect Genet Evol. 2018;61: 92–107. 10.1016/j.meegid.2018.03.015 29578082

[19] Čejková D, Zobaníková M, Chen L, Pospíšilová P, Strouhal M, Qin X, et al Whole genome sequences of three *Treponema pallidum ssp*. *pertenue* strains: yaws and syphilis treponemes differ in less than 0.2% of the genome sequence. PLoS Negl Trop Dis. 2012;6: e1471 10.1371/journal.pntd.0001471 PMC3265458 22292095

[20] Štaudová B, Strouhal M, Zobaníková M, Čejková D, Fulton LL, Chen L, et al 2014. Whole genome sequence of the *Treponema pallidum subsp*. *endemicum* strain Bosnia A: the genome is related to yaws treponemes but contains few loci similar to syphilis treponemes. PLoS Negl Trop Dis. 2014;8: e3261 10.1371/journal.pntd.0003261 25375929 PMC4222731

[21] Fraser CM, Norris SJ, Weinstock GM, White O, Sutton GG, Dodson R, et al Complete genome sequence of *Treponema pallidum*, the syphilis spirochete. Science 1998;281: 375–388. 9665876 10.1126/science.281.5375.375

[22] Giacani L, Jeffrey BM, Molini BJ, Le HT, Lukehart SA, Centurion-Lara A, et al Complete genome sequence and annotation of the *Treponema pallidum subsp pallidum* Chicago strain. J Bacteriol. 2010;192: 2645–2646. 10.1128/JB.00159-10 20348263 PMC2863575

[23] Giacani L, Iverson-Cabral SL, King JC, Molini BJ, Lukehart SA, Centurion-Lara A. Complete genome sequence of the *Treponema pallidum subsp*. *pallidum* Sea81-4 Strain. Genome Announc. 2014; 2: e00333–14. 10.1128/genomeA.00333-14 PMC3990758 24744342

[24] Matějková P, Strouhal M, Šmajs D, Norris SJ, Palzkill T, Petrosino JF, et al Complete genome sequence of *Treponema pallidum ssp*. *pallidum* strain SS14 determined with oligonucleotide arrays. BMC Microbiol. 2008;8: 76 10.1186/1471-2180-8-76 18482458 PMC2408589

[25] Pětrošová H, Zobaníková M, Čejková D, Mikalová L, Pospíšilová P, Strouhal M, et al Whole genome sequence of *Treponema pallidum ssp*. *pallidum*, strain Mexico A, suggests recombination between yaws and syphilis strains. PLoS Negl Trop Dis. 2012;6: e1832 10.1371/journal.pntd.0001832 23029591 PMC3447947

[26] Pětrošová H, Pospíšilová P, Strouhal M, Čejková D, Zobaníková M, Mikalová L, et al Resequencing of *Treponema pallidum ssp*. *pallidum* strains Nichols and SS14: correction of sequencing errors resulted in increased separation of syphilis treponeme subclusters. PLoS One. 2013;8: e74319 10.1371/journal.pone.0074319 24058545 PMC3769245

[27] Šmajs D, Zobaníková M, Strouhal M, Čejková D, Dugan-Rocha S, Pospíšilová P, et al Complete genome sequence of *Treponema paraluiscuniculi*, strain Cuniculi A: the loss of infectivity to humans is associated with genome decay. PLoS One. 2011;6: e20415 10.1371/journal.pone.0020415 21655244 PMC3105029

[28] Strouhal M, Mikalová L, Havlíčková P, Tenti P, Čejková D, Rychlík I, et al Complete genome sequences of two strains of *Treponema pallidum subsp*. *pertenue* from Ghana, Africa: Identical genome sequences in samples isolated more than 7 years apart. PLoS Negl Trop Dis. 2017;11: e0005894 10.1371/journal.pntd.0005894 28886021 PMC5607219

[29] Zobaníková M, Mikolka P, Čejková D, Pospíšilová P, Chen L, Strouhal M, et al Complete genome sequence of *Treponema pallidum* strain DAL-1. Stand Genomic Sci. 2012;10: 12–21. 10.4056/sigs.2615838 PMC3570794 23449808

[30] Arora N, Schuenemann VJ, Jäger G, Peltzer A, Seitz A, Herbig A, et al Origin of modern syphilis and emergence of a pandemic *Treponema pallidum* cluster. Nat Microbiol. 2016;2: 16245 10.1038/nmicrobiol.2016.245 27918528

[31] Pinto M, Borges V, Antelo M, Pinheiro M, Nunes A, Azevedo J, et al Genome-scale analysis of the non-cultivable *Treponema pallidum* reveals extensive within-patient genetic variation. Nat Microbiol. 2016;2: 16190 10.1038/nmicrobiol.2016.190 27748767

[32] Sun J, Meng Z, Wu K, Liu B, Zhang S, Liu Y, et al Tracing the origin of *Treponema pallidum* in China using next-generation sequencing. Oncotarget. 2016;7: 42904–42918. 27344187 10.18632/oncotarget.10154 PMC5189996

[33] Marks M., Fookes M., Wagner J., Butcher R., Ghinai R., Sokana O., Sarkodie Y.A., Lukehart S.A., Solomon A.W., Mabey D.C.W., Thomson N. 2018 Diagnostics for Yaws Eradication: Insights From Direct Next-Generation Sequencing of Cutaneous Strains of *Treponema pallidum*. Clin Infect Dis. 66:818–824. 10.1093/cid/cix892 29045605 PMC5848336

[34] Seshadri R, Myers GS, Tettelin H, Eisen JA, Heidelberg JF, Dodson RJ, et al Comparison of the genome of the oral pathogen *Treponema denticola* with other spirochete genomes. Proc Natl Acad Sci USA. 2004;101: 5646–5651. 10.1073/pnas.0307639101 15064399 PMC397461

[35] Walker EM, Arnett JK, Heath JD, Norris SJ. *Treponema pallidum subsp*. *pallidum* has a single, circular chromosome with a size of approximately 900 kilobase pairs. Infect Immun. 1991;59: 2476–2479. 2050412 10.1128/iai.59.7.2476-2479.1991 PMC258034

[36] Čejková D, Zobaníková M, Pospíšilová P, Strouhal M, Mikalová L, Weinstock GM, et al Structure of rrn operons in pathogenic non-cultivable treponemes: sequence but not genomic position of intergenic spacers correlates with classification of *Treponema pallidum* and *Treponema paraluiscuniculi* strains. J Med Microbiol. 2013;62: 196–207. 10.1099/jmm.0.050658-0 23082031 PMC3755535

[37] Centurion-Lara A, LaFond RE, Hevner K, Godornes C, Molini BJ, Van Voorhis WC, et al Gene conversion: a mechanism for generation of heterogeneity in the *tprK* gene of *Treponema pallidum* during infection. Mol Microbiol. 2004;52: 1579–1596. 10.1111/j.1365-2958.2004.04086.x 15186410

[38] Gray RR, Mulligan CJ, Molini BJ, Sun ES, Giacani L, Godornes C, et al Molecular evolution of the tprC, D, I, K, G, and J genes in the pathogenic genus *Treponema*. Mol Biol Evol. 2006;23: 2220–2233. 10.1093/molbev/msl092 16926243

[39] Giacani L, Brandt SL, Puray-Chavez M, Reid TB, Godornes C, Molini BJ, et al Comparative investigation of the genomic regions involved in antigenic variation of the TprK antigen among treponemal species, subspecies, and strains. J Bacteriol. 2012;194: 4208–4225. 10.1128/JB.00863-12 22661689 PMC3416249

[40] Čejková D, Strouhal M, Norris SJ, Weinstock GM, Šmajs D. A retrospective study on genetic heterogeneity within *Treponema* strains: subpopulations are genetically distinct in a limited number of positions. PLoS Negl Trop Dis. 2015;9: e0004110 10.1371/journal.pntd.0004110 26436423 PMC4593590

[41] Turner TB, Hollander DH. Biology of the treponematoses based on studies carried out at the International Treponematosis Laboratory Center of the Johns Hopkins University under the auspices of the World Health Organization. Monogr Ser World Health Organ. 1957;35: 3–266. 13423342

[42] Nichols HJ, Hough WH. Demonstration of Spirochaeta pallida in the cerebrospinal fluid. JAMA-J Am Med Assoc.1913;60: 108–110.

[43] Wendel GD Jr., Sanchez PJ, Peters MT, Harstad TW, Potter LL, Norgard MV. Identification of *Treponema pallidum* in amniotic fluid and fetal blood from pregnancies comlicated by congenital syphilis. Obstet Gynecol. 1991;78: 890–895. 1923218

[44] Stamm LV, Kerner TC Jr., Bankaitis VA, Bassford PJ Jr. Identification and preliminary characterization of *Treponema pallidum* protein antigens expressed in *Escherichia coli*. Infect Immun. 1983;41: 709–721. 6347894 10.1128/iai.41.2.709-721.1983 PMC264700

[45] Liska SL, Perine PL, Hunter EF, Crawford JA, Feeley JC. Isolation and transportation of *Treponema pertenue* in golden hamsters. Curr Microbiol. 1982;7: 41–43.

[46] Gastinel P, Vaisman A, Hamelin A, Dunoyer F. Study of a recently isolated strain of *Treponema pertenue*. Ann Dermatol Syphiligr Paris. 1963;90: 155–161. 13946771

[47] Turner TB, Hollander DH. Studies on treponemes from cases of endemic syphilis. Bull World Health Organ.1952;7: 75–81. 13019545 PMC2554131

[48] Tamura K, Stecher G, Peterson D, Filipski A, Kumar S. MEGA6: Molecular Evolutionary Genetics Analysis version 6.0. Mol Biol Evol. 2013;12: 2725–2729. 10.1093/molbev/mst197 PMC3840312 24132122

[49] Kimura M. A simple method for estimating evolutionary rate of base substitutions through comparative studies of nucleotide sequences. J Mol Evol. 1980;16: 111–120. 7463489 10.1007/BF01731581

[50] Tamura K. Estimation of the number of nucleotide substitutions when there are strong transition-transversion and G + C-content biases. Mol Biol Evol. 1992;9: 678–687. 10.1093/oxfordjournals.molbev.a040752 1630306

[51] Tamura K, Nei M. Estimation of the number of nucleotide substitutions in the control region of mitochondrial DNA in humans and chimpanzees. Mol Biol Evolution. 1993;10: 512–526. 10.1093/oxfordjournals.molbev.a040023 8336541

[52] Robinson DR, Foulds LR. Comparison of phylogenetic trees. Math Biosci. 1981;53: 131–147.

[53] Schliep K, Potts AJ, Morrison, DA, Grimm GW. Intertwining phylogenetic trees and networks. Meth Ecol Evol. 2017;8: 1212–1220.

[54] Nei M., Kumar S. Molecular evolution and phylogenetics. Oxford: Oxford University Press; 2000.

[55] Yang Z. PAML: a program package for phylogenetic analysis by maximum likelihood. Comput Appl Biosci. 1997;13: 555–556. 9367129 10.1093/bioinformatics/13.5.555

[56] Yang Z. PAML 4: Phylogenetic analysis by maximum likelihood. Mol Biol Evol. 2007;24: 1586–1591. 10.1093/molbev/msm088 17483113

[57] Xu B, Yang Z. PAMLX: a graphical user interface for PAML. Mol Biol Evol. 2013;30: 2723–2724. 10.1093/molbev/mst179 24105918

[58] Yang Z, Wong WSW, Nielsen R. Bayes empirical bayes inference of amino acid sites under positive selection. Mol Biol Evol.2005;22: 1107–1118. 10.1093/molbev/msi097 15689528

[59] Wong WSW, Yang Z, Goldman N, Nielsen R. Accuracy and power of statistical methods for detecting adaptive evolution in protein coding sequences and for identifying positively selected sites. Genetics. 2004;168: 1041–1051. 10.1534/genetics.104.031153 15514074 PMC1448811

[60] Yang Z, Nielsen R, Goldman N, Pedersen AMK. Codon-substitution models for heterogeneous selection pressure at amino acid sites. Genetics. 2000;155: 431–449. 10790415 10.1093/genetics/155.1.431 PMC1461088

[61] Yang Z, Swanson WJ, Vacquier VD. Maximum likelihood analysis of molecular adaptation in abalone sperm lysin reveals variable selective pressures among lineages and sites. Mol Biol Evol. 2000;17: 1446–1455. 10.1093/oxfordjournals.molbev.a026245 11018152

[62] Yang Z, Nielsen R. Codon-substitution models for detecting molecular adaptation at individual sites along specific lineages. Mol Biol Evol. 2002;19: 908–917. 10.1093/oxfordjournals.molbev.a004148 12032247

[63] Yang Z, dos Reis M. Statistical poperties of the branch-site test of positive selection. Mol Biol Evol. 2011;28: 1217–1228. 10.1093/molbev/msq303 21087944

[64] Kumar S, Stecher G, Li M, Knyaz C, Tamura K. MEGA X: Molecular Evolutionary Genetics Analysis across computing platforms. Mol Biol Evol. 2018;35: 1547–1549. 10.1093/molbev/msy096 29722887 PMC5967553

[65] Grillová L, Noda AA, Lienhard R, Blanco O, Rodríguez I, Šmajs D. Multilocus sequence typing of *Treponema pallidum subsp*. *pallidum* in Cuba from 2012 to 2017. J Infect Dis. 2018:10 16 10.1093/infdis/jiy604 [Epub ahead of print] 30325448

[66] Harper K.N., Ocampo P.S., Steiner B.M., George R.W., Silverman M.S., Bolotin S., et al 2008 On the origin of the treponematoses: a phylogenetic approach. PLoS Negl Trop Dis. 2: e148 10.1371/journal.pntd.0000148 18235852 PMC2217670

[67] Mikalová L, Strouhal M, Oppelt J, Grange PA, Janier M, Benhaddou N, et al Human *Treponema pallidum* 11q/j isolate belongs to subsp. *endemicum* but contains two loci with a sequence in TP0548 and TP0488 similar to subsp. *pertenue* and subsp. *pallidum*, respectively. PLoS Negl Trop Dis. 2017;11: e0005434 10.1371/journal.pntd.0005434 28263990 PMC5354452

[68] Strouhal M, Mikalová L, Haviernik J, Knauf S, Bruisten S, Oppelt J, et al Complete genome sequences of two strains of *Treponema pallidum subsp*. *pertenue* from Indonesia: modular structure of several treponemal genes. PLoS Negl Trop Dis. 2018;forthcoming. 10.1371/journal.pntd.0006867 PMC6197692 30303967

[69] Naqvi AA, Shahbaaz M, Ahmad F, Hassan MI. Identification of functional candidates amongst hypothetical proteins of *Treponema pallidum ssp*. *pallidum*. PLoS One. 2015;10: e0124177 10.1371/journal.pone.0124177 25894582 PMC4403809

[70] Radolf JD, Kumar S. The *Treponema pallidum* outer membrane. Curr Top Microbiol Immunol. 2017; 10.1007/82_2017_44 28849315 PMC5924592

[71] Centurion-Lara A, Giacani L, Godornes C, Molini BJ, Brinck Reid T, Lukehart SA. Fine analysis of genetic diversity of the tpr gene family among treponemal species, subspecies and strains. PLoS Negl Trop Dis. 2013;7: e2222 10.1371/journal.pntd.0002222 23696912 PMC3656149

[72] Wang G, Humayun MZ, Taylor DE. Mutation as an origin of genetic variability in *Helicobacter pylori*. Trends Microbiol. 1999;7: 488–493. 10603484 10.1016/s0966-842x(99)01632-7

[73] Radolf JD, Deka RK, Anand A, Šmajs D, Norgard MV, Yang XF. *Treponema pallidum*, the syphilis spirochete: making a living as a stealth pathogen. Nat Rev Microbiol. 2016;14: 744–759. 10.1038/nrmicro.2016.141 27721440 PMC5106329

[74] Grillová L, Bawa T, Gonzales F, Nieselt K, Mikalová L, Gayet-Ageron A, et al Molecular characterization of *Treponema pallidum subsp*. *pallidum* in Switzerland and France with a new multilocus sequence typing scheme. PLoS One. 2018;13: e0200773 10.1371/journal.pone.0200773 30059541 PMC6066202

[75] Pospíšilová P, Grange PA, Grillová L, Mikalová L, Janier M, Benhaddou N, et al Multi-locus sequence typing of *Treponema pallidum subsp*. *pallidum* present in clinical samples from France: infecting treponemes are genetically diverse and belong to 18 genotypes. PLoS One. 2018;13: e0201068 10.1371/journal.pone.0201068 30024965 PMC6053231

[76] Grange PA, Allix-Beguec C, Chanal J, Benhaddou N, Gerhardt P, Morini JP, et al Molecular subtyping of *Treponema pallidum* in Paris, France. Sex Transm Dis. 2013;40: 641–644. 10.1097/OLQ.0000000000000006 23859911

[77] Grange PA, Mikalová L, Gaudin C, Strouhal M, Janier M, Benhaddou N, et al *Treponema pallidum* 11qj subtype may correspond to a *Treponema pallidum subsp*. *endemicum* strain. Sex Transm Dis. 2016;43: 517–518. 10.1097/OLQ.0000000000000474 27419817

[78] Mikalová L, Strouhal M, Grillová L, Šmajs D. The molecular typing data of recently identified subtype 11q/j of *Treponema pallidum subsp*. *pallidum* suggest imported case of yaws. Sex Transm Dis. 2014;41: 552–553. 10.1097/OLQ.0000000000000165 25118969

[79] Mulligan CJ, Norris SJ, Lukehart SA. 2008. Molecular studies in *Treponema pallidum* evolution: toward clarity? PLoS Negl Trop Dis. 2008;2: e184 10.1371/journal.pntd.0000184 18357339 PMC2270795

[80] Lukehart SA, Giacani L. When is syphilis not syphilis? Or is it? Sex Transm Dis. 2014;41: 554–555. 10.1097/OLQ.0000000000000179 25118970 PMC4429752

